# Early Posttransplant urine ammonium-pH index levels and graft outcomes in kidney transplant recipients

**DOI:** 10.1007/s00424-026-03198-5

**Published:** 2026-07-24

**Authors:** Marie B. Nielsen, Nicoline V. Krogstrup, Mads V. Sørensen, Anders M. Kristensen, the CONTEXT Study Group*, Bente Jespersen, Mihai Oltean, Gertrude J. Nieuwenhuijs-Moeke, Frank J. M. F. Dor, Ina M. Schiessl, Jens Leipziger, Henrik Birn, Peder Berg

**Affiliations:** 1 Department of Renal Medicine, Aarhus University Hospital, Palle Juul-Jensens, Boulevard 35, 8200 Aarhus N Aarhus, Denmark; 2 Department of Clinical Medicine, Aarhus University Hospital, Aarhus, Denmark; 3 Department of Biomedicine, Aarhus University, Building 1115, Høegh-Guldbergs Gade 10, C. F. Møllers Allé 6, 8000 Aarhus C Aarhus, Denmark

**Keywords:** Kidney transplantation, Ammonium, PH, Tubular function, Kidney graft function

## Abstract

**Introduction:**

Functional assessment is essential to quantify kidney graft quality early after kidney transplantation. Kidney transplantation is inherently associated with ischemia–reperfusion-injury, a noxious insult for proximal tubule cells that may not be captured by glomerular function markers. The urine ammonium-pH index (uAPI) is a functional measure of the tubulointerstitial capacity for ammonium excretion. Here, we aimed to investigate the association between the uAPI and graft function in kidney transplant recipients.

**Methods:**

In this post-hoc observational analysis of the CONTEXT trial (NCT01395719), kidney transplant recipients with available urine samples were divided into an exploration cohort (n = 112) and a validation cohort (n = 88). The uAPI was assessed at baseline, 90 min after reperfusion, day 1, 2 and 3 posttransplant. Day 6 kidney graft biopsies were stained for VCAM1 as a marker of tubular injury. Outcomes were delayed graft function, estimated time to 50% reduction in plasma creatinine, and measured GFR on day 5 and 12 months posttransplant. Results were adjusted for age, sex, plasma creatinine and albuminuria.

**Results:**

In the exploration cohort, a higher uAPI on day 2 and day 3 was associated with lower VCAM1 expression (p < 0.001 and p = 0.002, respectively), lower risk of delayed graft function, and shorter estimated time to 50% reduction in plasma creatinine (p < 0.001). A higher uAPI on day 3 was associated with higher measured GFR on day 5 (p < 0.001) and at 12 months (p < 0.001) posttransplant. These findings were confirmed in the validation cohort and robust to adjustments. Associations between day 3 uAPI and long-term graft function were consistent in subgroup analyses, including in patients with vs. without delayed graft function. Inclusion of day 3 uAPI improved prediction of measured GFR > 60 at 12 months posttransplant.

**Conclusions:**

A higher uAPI likely informs about better tubulointerstitial function posttransplant and is associated with better early and 12-month kidney graft function.

**Supplementary Information:**

The online version contains supplementary material available at 10.1007/s00424-026-03198-5.

## Introduction

In many patients with kidney failure, kidney transplantation is the preferred treatment [1, 2]. Long-term graft survival is important for patients and for reducing organ shortage. Graft survival depends on multiple factors including donor organ quality and injury during the transplantation procedure, in part caused by ischemia–reperfusion-injury [3–5]. Early assessment of kidney graft quality by markers of glomerular injury (i.e., plasma creatinine and urine albumin-creatinine-ratio (uACR)) may fail to identify early and long-term impact of tubulointerstitial injury.

The urine ammonium-pH index (uAPI) was developed as a functional measure of the kidneys’ tubular capacity for ammonium excretion [6]. In patients with chronic kidney disease (CKD), a low uAPI associates with a markedly increased risk for CKD progression [6]. Urine ammonium excretion is a complex process that depends on both proximal tubular capacity for ammonium generation [7] and distal tubulo-interstitial function [8] for further transport and final excretion [9]. Proximal tubule cells are among the most energy consuming cells in the body and are very susceptible to damage by ischemia [10]. Kidney transplantation inherently entails variable ischemia–reperfusion-injury that may not be fully reflected by changes in plasma creatinine or albuminuria.

We hypothesized that the uAPI offers functional assessment of proximal tubular and tubulointerstitial damage to the graft that may provide additional information on the risk of early and long-term graft dysfunction defined by markers of early graft function including delayed graft function and measured glomerular filtration rate (mGFR) at 12 months.

## Methods

### Study design

The study is performed as a post-hoc analysis using the randomized controlled trial CONTEXT [11, 12] (ClinicalTrials.gov Identifier: NCT01395719, first submitted 14.07.2011). The CONTEXT study investigated the effect of pre-conditioning during deceased donor kidney transplantation. Adult kidney transplant recipients were included in the CONTEXT study between June 12, 2011, and December 28, 2014, and were followed for 12 months posttransplant. For exclusion criteria see Table S1. The study found no effect of pre-conditioning on early graft outcome [12], 12 months graft outcome [13] or selected biomarkers [14–16].

### Ethical approval and consent

The CONTEXT study was approved by the national data protection agencies and ethical committees (Denmark: The National Committee on Health Research Ethics; Sweden: Regional Ethical Board; the Netherlands: METCUMCG). The study was conducted in adherence with the Declaration of Helsinki. Informed and written consent was obtained from all participants.

### Exploration and validation cohort

The CONTEXT cohort (n = 222) was divided into an exploration cohort and a validation cohort. The exploration cohort included kidney transplant recipients from a single center in Aarhus (n = 131), Denmark. The internal validation cohort included kidney transplant recipients from multiple centers in Europe: Gothenburg (n = 46), Sweden; Groningen (n = 23) and Rotterdam (n = 22), the Netherlands. All recipients with available urine samples at (at least) one time point (baseline, 90 min, day 1, day 2 or day 3) were included in the present study.

### Data collection

Information regarding demographics and delayed graft function was collected from patient records. Information regarding donor type was obtained from ScandiaTransplant (for Aarhus and Gothenburg) and from Eurotransplant (for the Netherlands).

### Blood and urine sampling

Blood and urine were sampled immediately before kidney transplantation (baseline) and 90 min after reperfusion. After kidney transplantation, samples were collected on day 1, day 2, and day 3. In the exploration cohort, day 1 urine was collected 23 ± 9 h after reperfusion of the graft and a later sampling time within this period was associated with a higher uAPI (Figure S1). We therefore adjusted the associations between day 1 uAPI and outcomes for difference in time-to-sampling. Both urine and blood samples were handled within one hour, centrifugated at 2800G at 4 °C for ten minutes and stored at −70 to −80 °C.

### Biochemical analyses

Urine pH and urine ammonium were measured as previously described [6, 17, 18]. Urine pH was measured with a pH electrode (MetrOhm). Urine ammonium (NH_4_^+^) was measured with an NH_3_ selective gas membrane electrode (MetrOhm cat.no 60506150) with a pH-adjusting ionic strength adjuster according to manufacturer instructions. All samples were diluted 1:10 with demineralized water to ensure that ammonium concentration was within the linear detection range of the electrode. The uAPI was calculated as originally defined [6]:

$$uAPI=(log10([{{NH}_{4}}^{+}]) x {pH}^{3.6})/40$$

For uAPI calculations, the lower limit of ammonium was set to 1.1 mmol/L as previously described [6]. Thus, for samples with urine ammonium < 1.1 mmol/L, the concentration was defined as 1.1 mmol/L. Urease producing bacteria lead to a very alkaline urine with abnormally high ammonium levels because of urea splitting resulting in two NH_3_ per urea [19]. NH_3_ forms NH_4_^+^, resulting in a decrease in the [H^+^] and increase in [NH_4_^+^]. Consequently, samples with suspected contamination by urease producing bacteria were excluded as previously described (n = 2/707, 0.3%) [6].

Plasma and urine neutrophil gelatinase associated lipocalin (NGAL) were measured using a particle-enhanced turbidimetric immunoassay (BioPorto Diagnostics A/S, Hellerup, Denmark) at the Department of Clinical Biochemistry, Aarhus University Hospital. Urine chitinase-3-like protein 1 (YKL-40) and urine liver-type fatty acid-binding protein (L-FABP) were measured using sandwich ELISA (Bio-Techne, Minneapolis, USA; CMIC HOLDINGS Co., Ltd, Tokyo, Japan, respectively) [15].

Plasma creatinine, urine creatinine, and urine albumin were measured using automated, routine clinical assays at the local departments of clinical biochemistry.

### Kidney biopsy

Protocol biopsies were performed on day 6 after kidney transplantation. In the exploration cohort, a subset of the biopsies was stained for vascular cell adhesion molecule 1 (VCAM1) as previously described [20]. The biopsy staining was performed in kidney transplant recipients who were anuric pretransplant and had available urine samples on day 5 and 3 months posttransplant.

### Outcomes

Early graft outcomes were delayed graft function, estimated time to 50% reduction in plasma creatinine and mGFR on day 5. Delayed graft function was defined as need for dialysis within the first week posttransplant. The estimated time to 50% reduction in plasma creatinine was calculated using clinically available plasma creatinine samples as previously described [21]. In recipients requiring posttransplant dialysis, dialysis-induced decreases in plasma creatinine were not counted toward the 50% reduction. In kidney transplant recipients with primary non-function, the estimated time to 50% reduction could not be calculated. GFR was measured using ^51^Chrome-ethylenediamine tetraacetic acid (^51^Cr-EDTA) plasma clearance standardized to body surface area [22].

Longer term graft function was analyzed using mGFR at 12 months posttransplant. mGFR was assessed both as a continuous variable and dichotomously using a predefined cut-off of mGFR > 60 mL/min/1.73 m^2^.

### Missing data

An overview of the extent of missing data is shown in Table S2. No imputations were made, and data were analyzed using a complete case approach.

### Sample size calculation

After initial analyses in the exploration cohort, we calculated the sample size needed for validation of the association between the uAPI at day 3 and mGFR 12 months posttransplant. Using the association between the uAPI at day 3 posttransplant and mGFR 12 months posttransplant (slope: 1.18, SD uAPI: 7.7, SD mGFR: 19.4) a sample size of 40 kidney transplant recipients in the validation cohort would provide a power of 90% at an alpha of 5% to detect a significant association between day 3 uAPI and mGFR at 12 months posttransplant. These data were available for 51 out of 88 kidney transplant recipients in the validation cohort.

### Statistics

Stata/BE 19.5 (StataCorp) for Mac was used for statistical data analysis and graphical depiction of results. Data are presented as n (%) for dichotomic parameters. Continuous variables are presented as mean ± standard deviation (SD) if parametric and as median [Q1-Q3] if non-parametric.

Difference in the uAPI between donation after circulatory and brain death was assessed using a continuous mixed-effects model for repeated measures with uAPI as outcome, time point (day), donation-type and time-by-donation-type interaction as fixed effects, time as a random slope, participant as a random intercept and errors as an unstructured covariance matrix. Here, to allow a non-linear effect of time, time was included as a categorical variable (0, 1, 2, 3).

The uAPI was correlated to continuous outcomes (estimated time to 50% reduction in plasma creatinine and mGFR) using simple linear regression. Logarithmic transformation was used to obtain normal distribution for the estimated time to 50% reduction in plasma creatinine. The uAPI was associated with dichotomic outcomes (delayed graft function and mGFR > 60 mL/min/1.73 m^2^) using logistic regression. Multivariable analyses with adjustment for standard clinically available parameters (age, sex, plasma creatinine and uACR) were performed in the total cohort (exploration and validation combined). Subgroup analyses were performed using the ipdover command from the ipdmetan package [23].

All statistical testing was two-tailed and performed at a significance level of 5%.

## Results

### The exploration and validation cohort

Urine samples were available in 112 kidney transplant recipients in the exploration cohort and in 88 in the validation cohort. Baseline characteristics are provided in Table 1. Median uAPI was very low pretransplant (2.2 arbitrary units (a.u.) [1.9–6.3] in recipients on dialysis and 7.5 a.u. [6.0–9.4] in recipients not on dialysis in the exploration cohort, see Table 1). The uAPI pronouncedly increased posttransplant to 13.7 [10.4–17.5] on day 1 in the exploration cohort (Table 1). Posttransplant day 1–3 uAPI was lower in recipients receiving donations after circulatory death (−4.5 a.u., 95% CI: −7 to −2.1 to) compared to recipients receiving donations after brain death.

**Table 1:** Recipient characteristics, API and outcomes in the exploration cohort (n = 112) and validation cohort (n = 88). Data are n (%), mean ± standard deviation, or median [Q1-Q3]. A.u.: arbitrary units. NGAL: neutrophil gelatinase associated lipocalin. VCAM1: vascular cell adhesion molecule 1. L-FABP: liver-type fatty acid-binding protein. YKL-40: chitinase-3-like protein 1. mGFR: measured glomerular filtration rate. tCr50: estimated time to 50% reduction in P-creatinine. NA: not available

	Exploration cohort		Validation cohort	
*Recipient characteristics*	n		n	
Age, years	112	54 ± 12	88	60 ± 12
Sex, male	112	66 (59%)	88	50 (57%)
Pretransplant plasma-creatinine, µmol/L	112	639 ± 195	87	628 ± 236
Donortype				
Brain death, n		112 (100%)		68 (77%)
Circulatory death, n		0 (0%)		20 (23%)
*Urine ammonium-pH index*				
Pretransplant, a.u	56	2.5 [1.9–7.7]	44	2.6 [1.9–7.7]
In recipients on dialysis, a.u	40	2.2 [1.9–6.3]	37	2.6 [2–7.6]
In recipients not on dialysis, a.u	16	7.5 [6.0–9.4]	7	3.7 [1.5–15.5]
Posttransplant				
90 min, a.u	89	2.2 [1.7–7.5]	35	2.4 [2.0–10.3]
Day 1, a.u	83	13.7 [10.4–17.5]	75	9.1 [6.9–13.0]
Day 2, a.u	61	11.1 [9.6–15.9]	78	9.9 [7.6–13.8]
Day 3, a.u	88	17.0 [12.5–21.7]	75	10.5 [8.2–15.7]
*Tubular damage*				
Biopsy VCAM1, % tubular area				
Day 6	26	3.3 [1.7–10.8]	NA	NA
Plasma NGAL, µg/L				
Day 1	82	338 [219–478]	71	430 [255–552]
Day 3	88	249 [174–367]	70	278 [151–497]
Urine NGAL, µg/g				
Day 1	83	389 [185–982]	70	831 [294–1941]
Day 3	87	354 [122–993]	68	355 [87–1225]
Urine L-FABP, µg/g				
Day 1	83	66 [40–118]	72	96 [54–305]
Day 3	87	47 [27–84]	68	57 [25–86]
Urine YKL-40, µg/g				
Day 1	83	49 [6–127]	72	47 [8–195]
Day 3	87	11 [2–41]	67	7 [1–100]
*Early graft outcomes*				
Primary non-function, n	112	4 (4%)	88	5 (6%)
Delayed graft function, n	112	34 (30%)	88	35 (40%)
tCr50, days	108	4.2 [1.2–8.2]	83	6.8 [2.7–11.2]
mGFR day 5, mL/min/1.73 m^2^	68	34 [24–48]	21	22 [17–43]
*12 months graft outcomes*				
mGFR, mL/min/1.73 m^2^	54	49 ± 20	50	49 ± 21
mGFR > 60 mL/min/1.73 m^2^	54	16 (30%)	50	12 (24%)

### Posttransplant tubular injury and integrity

**Fig. 1:**
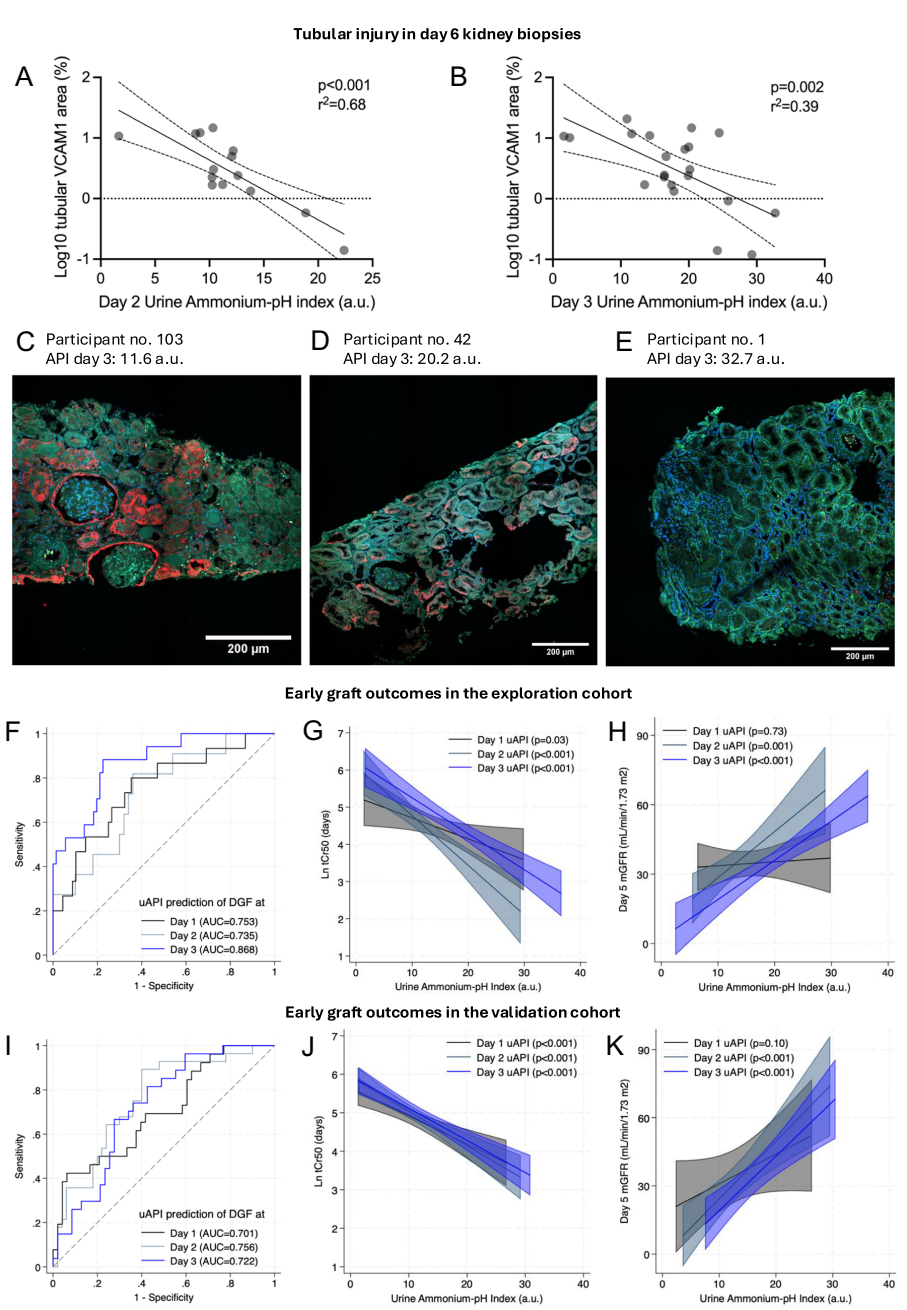
Tubular injury and early graft outcomes. **A-B**) Day 6 biopsy tubular VCAM1 expression as a function of the urine ammonium-pH index (uAPI) at day 2 (A, n = 14) and day 3 (B, n = 21) in a subset of the exploration cohort. C-E) Tubular VCAM1 day 6 (red) in three different participants with different day 3 urine ammonium-pH index levels (blue: nuclei (Hoechst); green: tubular autofluorescence). Delayed graft function (DGF), estimated time to 50% reduction in plasma creatinine (tCr50), and mGFR day 5 in F–H) the exploration cohort and I-K) the validation cohort. In the exploration cohort, day 1 urine was collected 23 ± 9 h after reperfusion of the graft and a later sampling time within this period was associated with a higher uAPI (Figure S1). We therefore adjusted the associations between day 1 uAPI and outcomes for difference in time-to-sampling. uAPI: urine ammonium-pH index. VCAM1: vascular cell adhesion molecule 1

VCAM1 is a marker of proximal tubular epithelial cells that have failed to repair after injury, and a higher VCAM1 expression is associated with increased risk of acute kidney injury to CKD transition [20, 24]. In the exploration cohort, a higher uAPI at day 2 and day 3 was associated with lower tubular VCAM1 expression at day 6 posttransplant (Fig. 1A-E). Furthermore, a higher day 1, 2, and 3 uAPI were associated with lower plasma NGAL concentrations (Figure S2), and a higher day 3 uAPI was associated with lower levels of urinary markers of tubular injury (L-FABP, YKL-40, and NGAL) in both cohorts (Figure S2).

These results substantiate the hypothesis that early uAPI measurements report on the tubular integrity and associate with the degree of tubular damage in kidney transplant recipients.

### Early graft outcomes

A higher uAPI was associated with better early graft outcomes in both cohorts. A higher uAPI at day 1, 2, and 3 were associated with a lower risk of delayed graft function (Fig. 1F + I) and a shorter estimated time to 50% reduction of plasma creatinine (Fig. 1G + J). A higher uAPI on day 2 and 3 were associated with higher mGFR at day 5 posttransplant (Fig. 1H + K).

All findings were robust to adjustments for age, sex, plasma creatinine and uACR in the pooled cohort (exploration and validation cohort combined, Table 2). On day 1, a 1 SD higher uAPI was associated with an adjusted 0.46-fold lower odds of delayed graft function and a 19% shorter estimated time to 50% reduction of plasma creatinine (Table 2). A combined model with donor age, sex, plasma creatinine and uACR predicted delayed graft function with an AUC of 0.923 and was marginally elevated by further addition of day 1 uAPI (AUC of 0.937, p = 0.17, Figure S3). Net reclassification improvement measures to what degree a new model/measure correctly changes the predicted risk of individuals, while integrated discrimination improvement quantifies the overall improvement in risk prediction by comparing differences in predicted probabilities between individuals with and without events. Inclusion of the uAPI resulted in a net reclassification improvement of 36% (p = 0.051) and in an integrated discriminatory improvement of 2% (p = 0.24) (Table 3).

**Table 2:** Crude and adjusted associations between API and graft outcomes in the pooled cohort (exploration and validation cohort combined). *Adjusted for age (years), sex, plasma creatinine (µmol/L) and urine albumin creatinine ratio (mg/g). #Only measured in Aarhus and Gothenburg. uAPI: urine ammonium-pH index. SD: standard deviation. A.u.: arbitrary units. tCr50: estimated time to 50% reduction in plasma creatinine. mGFR: measured glomerular filtration rate. N.A.: not analyzed

	uAPI, per SD higher		
*Early graft outcomes*	Day 1 (SD: 6 a.u.)	Day 2 (SD: 5.5 a.u.)	Day 3 (SD: 7.3 a.u.)
Delayed graft function, OR (95% CI)			
Crude Adjusted*	0.32 (0.19 to 0.54) 0.46 (0.23 to 0.92)	0.26 (0.14 to 0.48) N.A	0.18 (0.10 to 0.34) 0.41 (0.18 to 0.90)
tCr50, % (95% CI)			
Crude Adjusted*	−32% (−43 to −19) −19% (−31 to −4)	−45% (−53 to −36) N.A	−49% (−57 to −42) −29% (−41 to −16)
Day 5 mGFR, mL/min/1.73 m^2^ (95% CI)#			
Crude Adjusted*	3.0 (−1.4 to 7.3) 0.7 (−3 to 4.4)	12.4 (7.9 to 16.9) N.A	13.2 (9.4 to 17.1) 5.0 (1.2 to 8.8)
*12 months graft outcomes*			
mGFR, mL/min/1.73 m^2^ (95% CI)			
Crude Adjusted*	N.A N.A	N.A N.A	7.9 (4.1 to 11.7) 5.4 (0.7 to 10.1)
mGFR > 60 mL/min/1.73 m^2^, OR (95% CI)			
Crude Adjusted*	N.A N.A	N.A N.A	2.4 (1.5 to 4) 2.7 (1.3 to 5.8)

**Table 3:** Net reclassification improvement and integrated discriminatory improvement of the urine ammonium-pH index. Net reclassification improvement and integrated discriminatory improvement by adding the urine ammonium-pH index to a model including recipient age, sex, plasma creatinine and urine albumin-creatinine-ratio at day 1 (for delayed graft function) or day 3 (for mGFR > 60 ml/min/1.73m^2^). uAPI: urine ammonium-pH index. mGFR: measured glomerular filtration rate

*Net Reclassification Improvement by the uAPI*	Patients without events	Patients with events	Total
Delayed graft function, % (95% CI)	18.3	17.9	36.2 (0.0 to 73)
12-month mGFR > 60, % (95% CI)	24.3	21.4	45.8 (2.3 to 89)
*Integrated discriminatory improvement by the uAPI*			
Delayed graft function, % (95% CI)	2.0 (−1.3 to 5.3)		
12-month mGFR > 60, % (95% CI)	7.3 (1.5 to 13.2)		

We also assessed an alternative model based on available donor and recipient characteristics associated with increased risk of delayed graft function [5, 25] (donor age, terminal donor creatinine, donation type, cold ischemia time, and recipient diabetes status (yes/no), BMI, and dialysis pre-transplant (yes/no). Kidney donor profile index (KDPI) was not available. Here, AUC increased from 0.792 to 0.865 (p = 0.038) by inclusion of day 1 uAPI (Figure S4). Net reclassification improvement was 47% (p = 0.03) and integrated discriminatory improvement was 10% (p = 0.004) (Table S3).

### Graft outcomes 12 months posttransplant

**Fig. 2:**
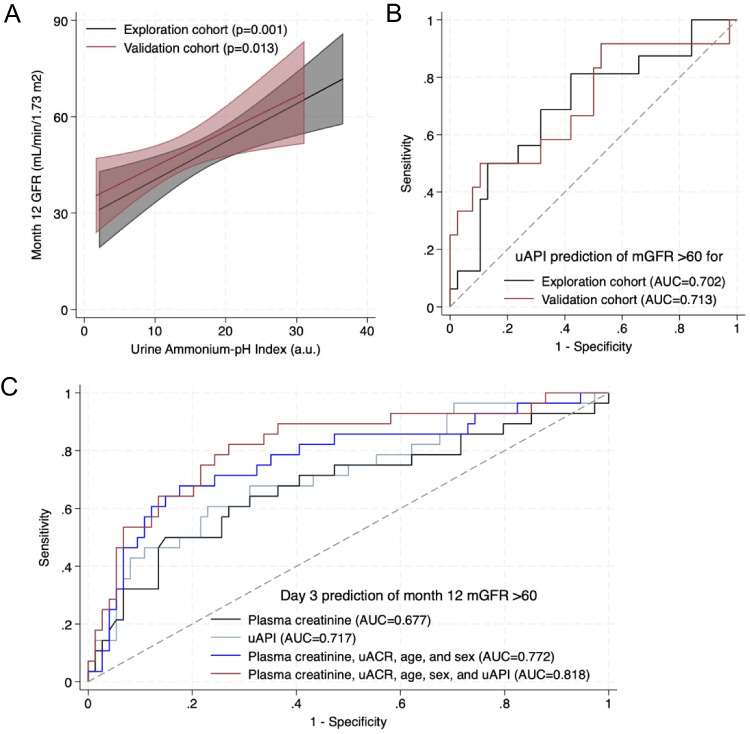
Graft outcomes 12 months posttransplant. **A**) The association between day 3 urine ammonium-pH index (uAPI) and 12-month mGFR and **B**) prediction of 12 months mGFR > 60 mL/min/1.73 m^2^ in the exploration (black) and validation cohort (red). **C**) Prediction of mGFR > 60 mL/min/1.73 m^2^ in the combined cohort (exploration and validation). uAPI: urine ammonium-pH index. mGFR: measured glomerular filtration rate. uACR: urine albumin-creatinine ratio

A higher uAPI on day 3 was associated with higher mGFR and a higher odds of having mGFR above > 60 mL/min/1.73 m^2^ 12 months after kidney transplantation in both cohorts (Fig. 2A-B). These findings were robust to adjustments for age, sex, plasma creatinine and uACR (Table 2). In the combined cohort, a 1 SD higher uAPI at day 3 was associated with an adjusted 5.4 mL/min/1.73 m^2^ (95% CI: 0.7–10.1) higher mGFR 12 months posttransplant.

In subgroup analyses, a higher day 3 uAPI was consistently associated with a higher mGFR at 12 months posttransplant across patient sex, donor sex, patients with or without delayed graft function and patients on or off dialysis pretransplant (Figure S5). Also, associations were robust to further adjustment for delayed graft function (yes vs. no) and estimated time to 50% reduction in plasma creatinine (Table S4).

A combined model with day 3 age, sex, plasma creatinine and uACR predicted mGFR > 60 with an AUC of 0.772 (vs. 0.677 for day 3 plasma creatinine alone) and was elevated further by day 3 uAPI (AUC of 0.818, Fig. 2C). Inclusion of the uAPI resulted in a net reclassification improvement of 46% (p = 0.039) and integrated discriminatory improvement of 7% (p = 0.014) (Table 3).

## Discussion

The uAPI was developed to provide a measure of the tubular capacity for ammonium generation and excretion independent of the systemic need for acid excretion [6]. As intact proximal tubular and distal tubulointerstitial function is a pre-requisite for appropriate ammonium excretion, the uAPI could potentially be an indicator of functional tubulointerstitial health/integrity. As these compartments, especially the proximal tubules, are very susceptible to ischemia–reperfusion-injury, uAPI measurements could be valuable in a kidney transplant setting. This prospective, observational study investigated the uAPI as an early marker of graft function in kidney transplant recipients.

Before kidney transplantation, the uAPI level was lower compared to measurements in patients with CKD stage 3–4 [6]. In a previous study, we found an uAPI of ~ 23 a.u. in healthy controls. In the CONTEXT cohort, the median uAPI was 7.5 a.u. in patients who were not on dialysis before kidney transplantation and 2.2 a.u. in patients on long-term dialysis compared to a median uAPI of 12.1 and 10.3 in CKD stage 3 and 4 patients, respectively. This illustrates a progressive decline in kidney function that is also seen by the declining uAPI.

The uAPI increased markedly after kidney transplantation, although with large variability (high SDs). This could indicate large heterogeneity in tubulointerstitial health and/or the degree of transplant-associated damage in the transplanted kidney grafts. In congruence with this hypothesis, a higher posttransplant uAPI was associated with lower levels of tubular injury markers, less proximal tubular injury on day 6, and better graft outcomes at early time points and 12 months posttransplant in the exploration and the validation cohort.

Higher uAPI was associated with better early graft outcomes. This indicates that including the uAPI to assess tubulointerstitial integrity/the extent of damage posttransplant provides additional information on early graft function beyond what is captured by plasma creatinine and uACR. Hence, it may be useful as an early marker of graft function. Inclusion of day 1 uAPI to standard clinical variables yielded borderline statistically significant improvements in classification of a similar magnitude to what albuminuria provides for the prediction of kidney failure in CKD (36% vs. 25–44%) [26].

A higher uAPI on day 3 was associated with better 12 months graft function. Importantly, this association was consistent in patients with and in patients without delayed graft function and robust to adjustment for delayed graft function (yes vs. no) and estimated time to 50% reduction in plasma creatinine. These findings illustrate that the uAPI informs about long-term graft function independently of plasma creatinine, uACR and the occurrence of delayed graft function. Furthermore, inclusion of the day 3 uAPI to standard clinical variables (age, sex, plasma creatinine and uACR) improved the prediction of mGFR > 60 mL/min/1.73 m^2^ 12 months posttransplant.

Different approaches have been used to interrogate tubular function/injury in the early posttransplant period. Urinary cystatin C on day 1–2 after transplantation was higher in patients with delayed graft function and associated with eGFR at three months [27]. Urine and plasma NGAL were found to be associated with early graft function, but neither plasma NGAL nor urine NGAL, cystatin C, L-FABP or YKL-40 associated with 12-month graft function [15]. Further, structural indices of proximal tubular injury are detectable within an hour of reperfusion as loss of Na⁺/K⁺-ATPase polarity [28], and on early (day 10) biopsies a higher staining-positive area of KIM-1 associated with a lower number of functioning proximal tubular epithelial cells [29], but not consistently with delayed graft function or long-term graft function [20, 29]. Our findings are consistent with these observations, the uAPI associated with tubular VCAM1 expression and injury markers, and extended them by providing a functional, urine-based readout of the tubule's capacity to generate and excrete ammonium, rather than a marker of structural damage.

To our knowledge, tubular acid/ammonium handling has not previously been examined as an early prognostic measure after kidney transplantation. However, beyond the immediate posttransplant period, impaired renal acid handling has repeatedly been linked to graft outcomes. Metabolic acidosis is common after transplantation and, in observational cohorts, a lower serum bicarbonate is associated with a higher risk of graft loss and death. In a multicenter cohort of 2318 recipients, a low total CO₂ three months after transplantation was associated with an increased risk of graft loss [30], and higher serum bicarbonate has been associated with better graft and patient survival [31]. Notably, the same axis is implicated when acid handling is assessed: in the TransplantLines cohort, a higher dietary acid load was associated with increased risk of graft failure. The association was partly mediated by lower venous bicarbonate, whereas a higher urinary ammonium excretion attenuated the risk conferred by a high acid load [32]. The capacity to mount an ammonium response thus buffered the harm of acid retention, mirroring the findings in native kidney CKD patients that low urinary ammonium and net acid excretion is associated with CKD progression and death [33–35].

The interpretation that metabolic acidosis itself drives these outcomes is also challenged by interventional data. In the Preserve-Transplant trial, two years of sodium bicarbonate supplementation effectively corrected metabolic acidosis but did not slow the decline in estimated GFR in stable transplant recipients [36]. The dissociation, that acidosis is linked to adverse outcomes observationally, but its pharmacological correction confers no benefit, could suggest that a low serum bicarbonate may largely be a marker of underlying tubulointerstitial dysfunction rather than the cause of progressive graft decline. Whether selecting or monitoring recipients by tubular function, e.g. with the uAPI, can identify those in whom kidney-protective strategies (e.g. SGLT2 inhibitor treatment) warrants prospective investigations.

The present study is strengthened by the prospective, multicenter, and multinational design of the CONTEXT study. Also, mGFR measurements and VCAM1 biopsy staining strengthen the study by providing a reasonably precise estimate of posttransplant graft filtration function and tissue level injury. Furthermore, the validation cohort included both donations after brain death and circulatory death. No living donor donations were included in the CONTEXT cohort, and our results cannot be directly extrapolated to this setting without further investigations including this donation type.

The study is limited by variation in time points of sampling on day 1, missing plasma creatinine and uACR on day 2, missing mGFR measurements on day 5 and 12 months and the post-hoc nature of the study. As the uAPI is measured in urine, the results are limited to kidney transplant recipients with urine production posttransplant. Further validation of the usefulness of the uAPI in kidney transplant recipients is needed.

In conclusion, a higher uAPI in the first days posttransplant is associated with better graft outcomes. It may be valuable as a measure of early graft function and tissue level injury, and its inclusion may improve models for predicting graft function. In kidney transplant recipients with urine production, the uAPI is suggested to be a useful measure of tubular function.

## Supplementary Information

Below is the link to the electronic supplementary material.

Supplementary file1 (PDF 1318 KB)

## Data Availability

Individual-level clinical data used in this study cannot be made publicly available, as participant consent for data sharing was not obtained. Researchers who would like to inspect the raw data may apply for controlled access, subject to institutional approval. Data inquiries should be directed to the corresponding authors.

## Declarations

### Data sharing statement

Individual-level clinical data used in this study cannot be made publicly available, as participant consent for data sharing was not obtained. Researchers who would like to inspect the raw data may apply for controlled access, subject to institutional approval. Data inquiries should be directed to the corresponding authors.
