# Supplementary material for: Early Posttransplant urine ammonium-pH index levels and graft outcomes in kidney transplant recipients
Source: Pflugers Arch. 2026 Jul 24;478(8):68. doi: 10.1007/s00424-026-03198-5 (PMC13395979; doi:10.1007/s00424-026-03198-5)
Supplement: Supplementary file 1 — Supplementary file1 (PDF 1318 KB) [file 424_2026_3198_MOESM1_ESM.pdf]

Supplemental Material for:

Early Posttransplant Urine Ammonium-pH Index Levels and Graft Outcomes in Kidney Transplant Recipients

[1,2,#]Marie B. Nielsen, [1]Nicoline V. Krogstrup, [3]Mads V. Sørensen, [3]Anders M. Kristensen, the CONTEXT Study Group*, [3]Ina M. Schiessl, [3]Jens Leipziger, [1,2]Henrik Birn, [3,#]Peder Berg

[1]Department of Renal Medicine, Aarhus University Hospital, Aarhus, Denmark

[2]Department of Clinical Medicine, Aarhus University Hospital, Aarhus, Denmark

[3]Department of Biomedicine, Aarhus University, Aarhus, Denmark

*The CONTEXT study group is listed in Acknowledgments

[#]Correspondence:

Marie Bodilsen Nielsen, Palle Juul-Jensens Boulevard 35, 8200 Aarhus N, Denmark, marie.bodilsen@clin.au.dk

Peder Berg, Building 1115, Høegh-Guldbergs Gade 10, C. F. Møllers Allé 6, 8000 Aarhus, peder.berg@biomed.au.dk

Table of Contents

Table of Contents....2
Supplementary Tables....3
Table S1: Exclusion criteria for the CONTEXT study....3
Table S2: Extent of missing data of covariates....4
Table S3: Net reclassification improvement and integrated discriminatory improvement of the urine ammonium-pH index added to a model including donor and recipient related risk factors for delayed graft function....5
Table S4: Sensitivity analyses of the association between API and 12 months graft outcomes....6
Supplementary Figures....7
Figure S1: Association between sampling time and day 1 API....7
Figure S2: Association between day 1-3 API and tubular injury markers....8
Figure S3: Association with delayed graft function, model 1....10
Figure S4: Association with delayed graft function, model 2....11
Figure S5: Subgroup analyses of API and 12-month graft function....12

Supplementary Tables

Table S1: Exclusion criteria for the CONTEXT study

Exclusion criteria
1. Arteriovenous fistula in the leg of planned remote ischemic conditioning
2. Pre-existing lower limb ischemia
3. Other immunosuppression than standard regimen
4. Double kidney transplantation

Table S2: Extent of missing data of covariates

**Missing data**	Exploration cohort	Validation cohort
Covariates	n (%)	n (%)
Age	0 (0)	0 (0)
Sex	0 (0)	0 (0)
Plasma creatinine		
Day 1	0 (0)	1 (1)
Day 3	0 (0)	0 (0)
Urine albumin creatinine ratio		
Day 1	0 (0)	2 (3)
Day 3	1 (1)	3 (4)
With day 1 uAPI available:		
Donor age	0 (0)	0 (0)
Donor terminal creatinine	12 (14)	7 (9)
Cold ischemia time	16 (19)	5 (7)
Donation type	0 (0)	0 (0)
Pretransplant dialysis status	0 (0)	0 (0)
Pretransplant BMI	6 (7)	15 (20)
Pretransplant diabetes status	0 (0)	0 (0)
Early graft outcomes		
DGF with uAPI available at:		
Day 1	0 (0)	0 (0)
Day 2	0 (0)	0 (0)
Day 3	0 (0)	0 (0)
tCr50 with uAPI available at:		
Day 1	1 (1)	4 (5)
Day 2	1 (2)	2 (3)
Day 3	2 (2)	2 (3)
mGFR day 5 with uAPI available at:		
Day 1	32 (39)	59 (79)
Day 2	26 (43)	60 (77)
Day 3	31 (35)	56 (75)
12 months graft outcomes		
mGFR with uAPI available at:		
Day 3	34 (39)	24 (32)

Table S2: Extent of missing data of covariates
Extent of missing data for covariates and outcomes in the exploration and validation cohort with available API measurements. DGF: delayed graft function. uAPI: urine ammonium-pH index. tCr50: estimated time to 50% reduction in plasma creatinine. mGFR: measured glomerular filtration rate.

Table S3: Net reclassification improvement and integrated discriminatory improvement of the urine ammonium-pH index added to a model including donor and recipient related risk factors for delayed graft function

Net Reclassification Improvement by the uAPI in:	Patients without events	Patients with events	Total
Delayed graft function, % (95% CI)	20.5	26.7	47.2 (4.7 to 90)
	Integrated discriminatory improvement by the uAPI		
Delayed graft function, % (95% CI)	10.2 (3.2 to 17.2)		

Table S3: Net reclassification improvement and integrated discriminatory improvement of the urine ammonium-pH index added to a model including donor and recipient related risk factors for delayed graft function

Net reclassification improvement and integrated discriminatory improvement by adding the urine ammonium-pH index to a model including donor age, donor terminal creatinine, donation type, cold ischemia time, and recipient diabetes status (yes/no), BMI, and dialysis pre-transplant (yes/no). Kidney donor profile index (KDPI) was not available. uAPI: urine ammonium-pH index.

Table S4: Sensitivity analyses of the association between API and 12 months graft outcomes

	API, per SD higher		
12 months graft outcomes	Day 1 (6 a.u.)	Day 2 (5.5 a.u.)	Day 3 (7.3 a.u.)
mGFR, mL/min/1.73 m^2 (95% CI)			
Crude	N.A.	N.A.	7.9 (4.1 to 11.7)
Model 1*			5.5 (0.9 to 10.1)
Model 2#			4.4 (0.3 to 8.8)
mGFR > 60 mL/min/1.73 m^2, OR (95% CI)			
Crude	N.A.	N.A.	2.4 (1.5 to 4)
Model 1*			2.7 (1.3 to 5.8)
Model 2#			2.4 (1.1 to 5)

Table S4: Sensitivity analyses of the association between API and 12 months graft outcomes.
*Adjusted for age (years), sex, p-creatinine, DGF (yes vs. no), and urine albumine creatinine ratio (mg/g). #Adjusted for age (years), sex, DGF (yes vs. no), time to 50% p-creatinine reduction, and urine albumine creatinineratio (mg/g). API: ammonium-pH index. SD: standard deviation. A.u.: arbitrary units. N.A.: not analyzed. DGF: delayed graft function. mGFR: measured glomerular filtration rate. AP index: urine ammonium-pH index.

Supplementary Figures

Figure S1: Association between sampling time and day 1 API

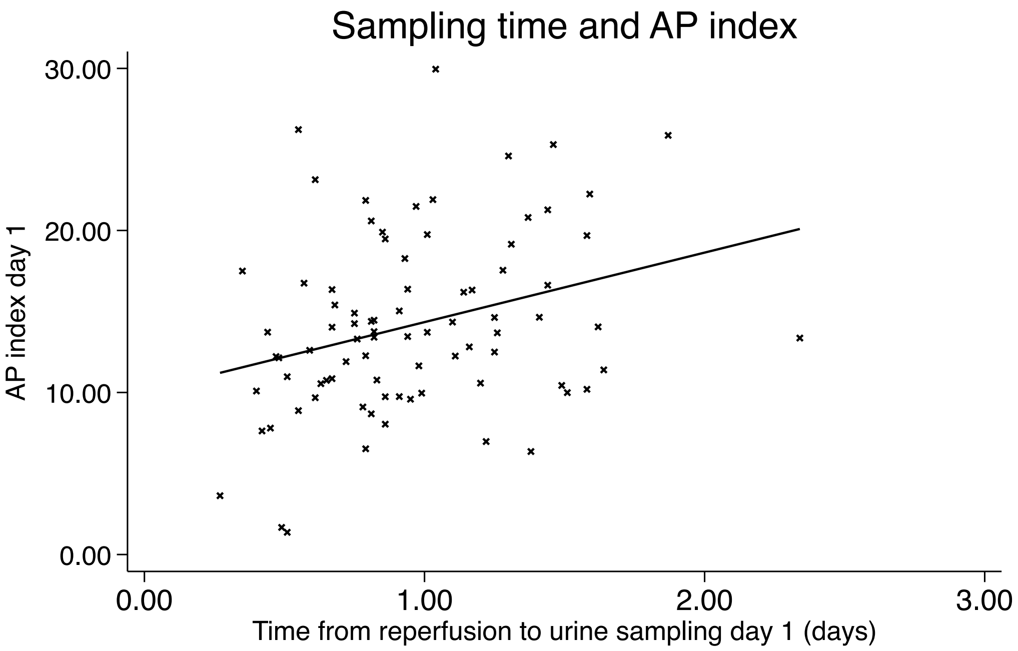

Figure S1: Association between time point of sampling and AP index day 1 in the exploration cohort. AP index: urine ammonium-pH index.

Figure S2: Association between day 1-3 API and tubular injury markers

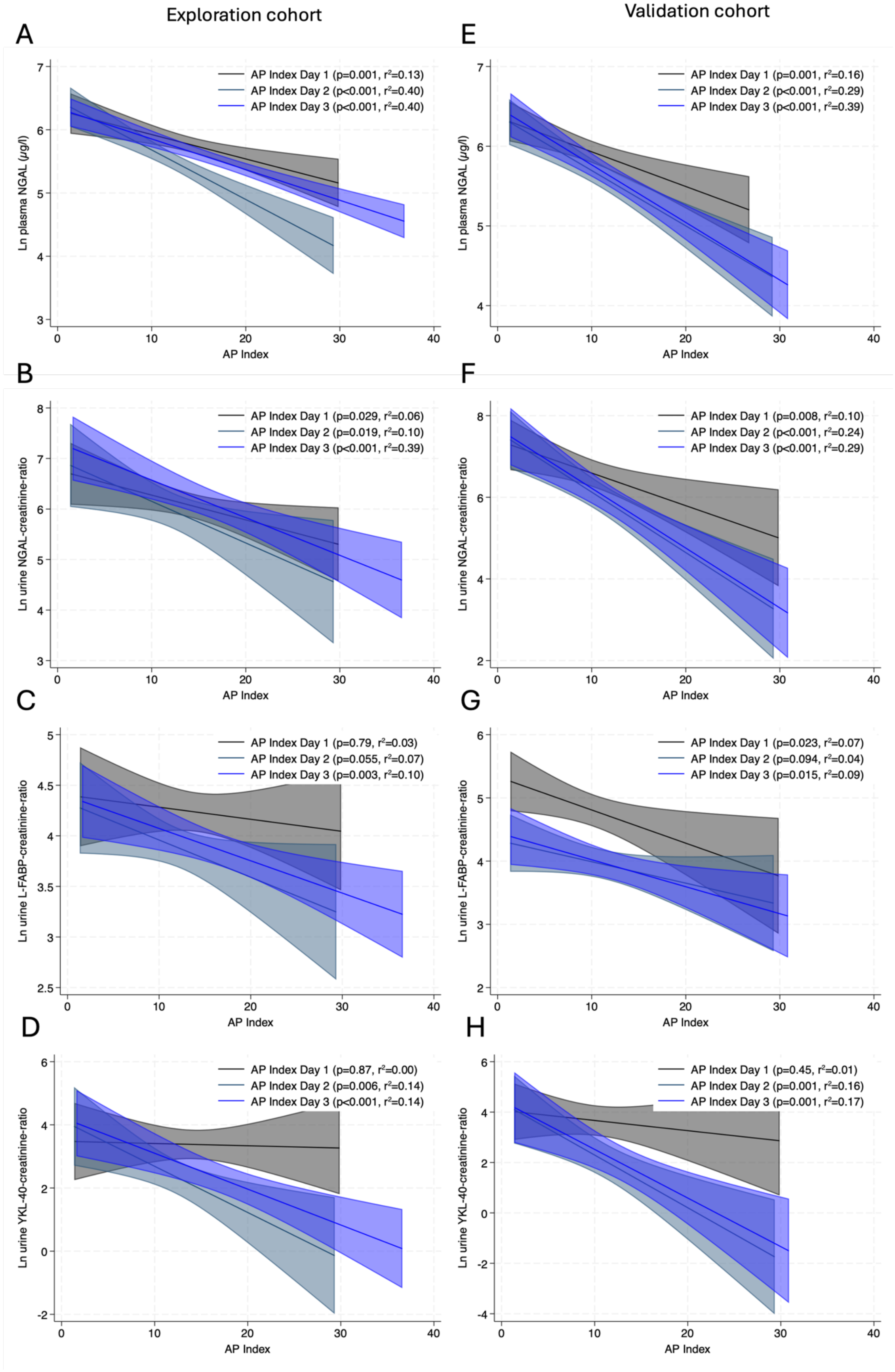

Figure S2: Plasma and urine biomarkers. Association between day 1-3 API and plasma NGAL, urine NGAL-creatinine-ratio, L-FABP-creatinine-ratio and YKL-40-creatinine-ratio in A-D) the exploration cohort and E-H) the validation cohort. Associations were assessed between day 1 API and day 1 damage markers whereas API at day 2 and 3 API were associated with day 3 damage markers.
API: urine ammonium-pH index. NGAL: neutrophil gelatinase-associated lipocalin. L-FABP: liver-type fatty acid-binding protein. YKL-40: chitinase-3-like protein 1.

Figure S3: Association with delayed graft function, model 1

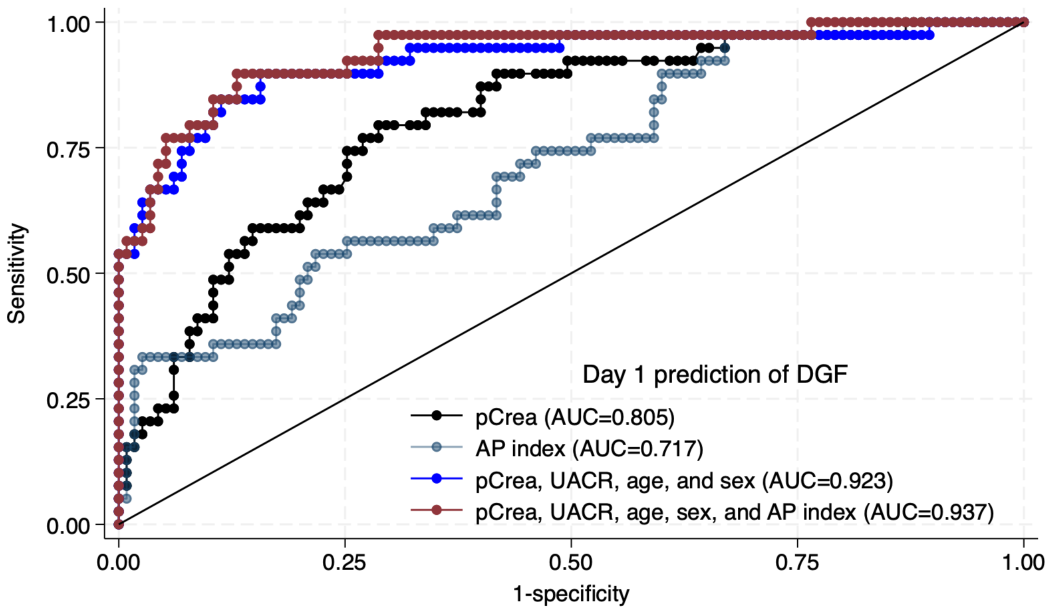

Figure S3: Prediction of delayed graft function (DGF) in the combined cohort (exploration and validation) at day 1 after kidney transplantation. AP index: urine ammonium-pH index.

Figure S4: Association with delayed graft function, model 2

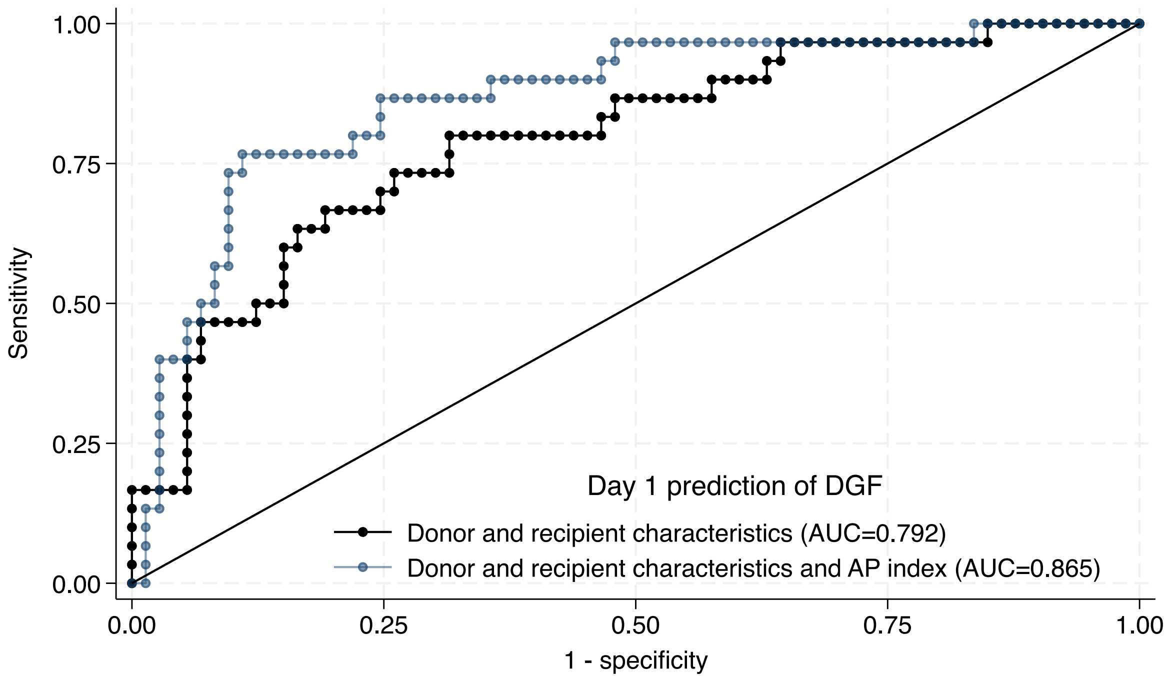

Figure S4: Prediction of delayed graft function (DGF) in the combined cohort (exploration and validation) at day 1 after kidney transplantation. Donor and recipient characterstics included were: donor age, terminal creatinine, donation type, cold ischemia time, and recipient diabetes status (yes/no), BMI, and dialysis pre-transplant (yes/no). Kidney donor profile index (KDPI) was not available. AP index: urine ammonium-pH index.

Figure S5: Subgroup analyses of API and 12-month graft function

A) mGFR at 12 months posttransplant per 1 SD higher day 3 API

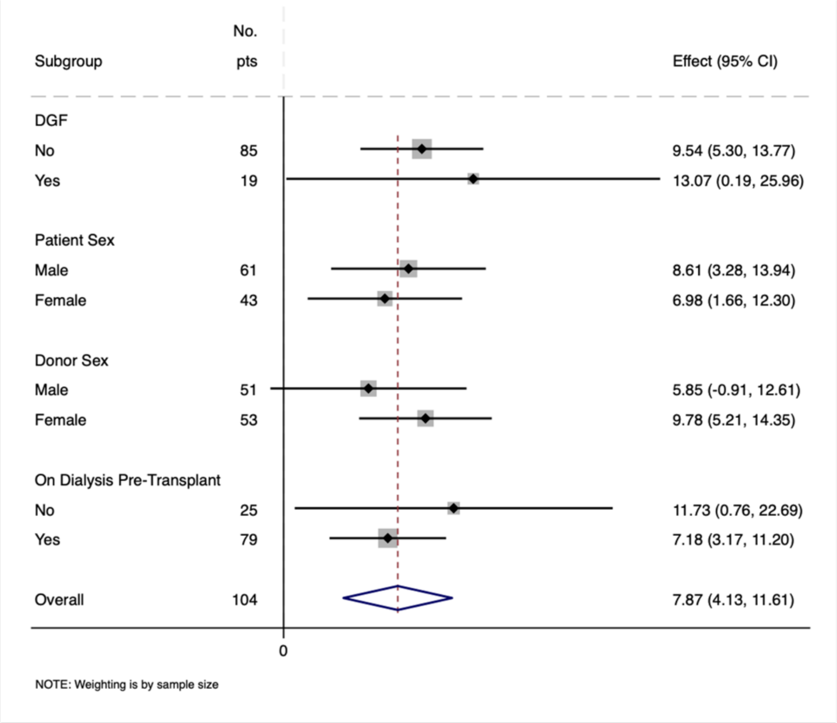

B) Odds for mGFR > 60 at 12 months posttransplant per 1 SD higher day 3 API

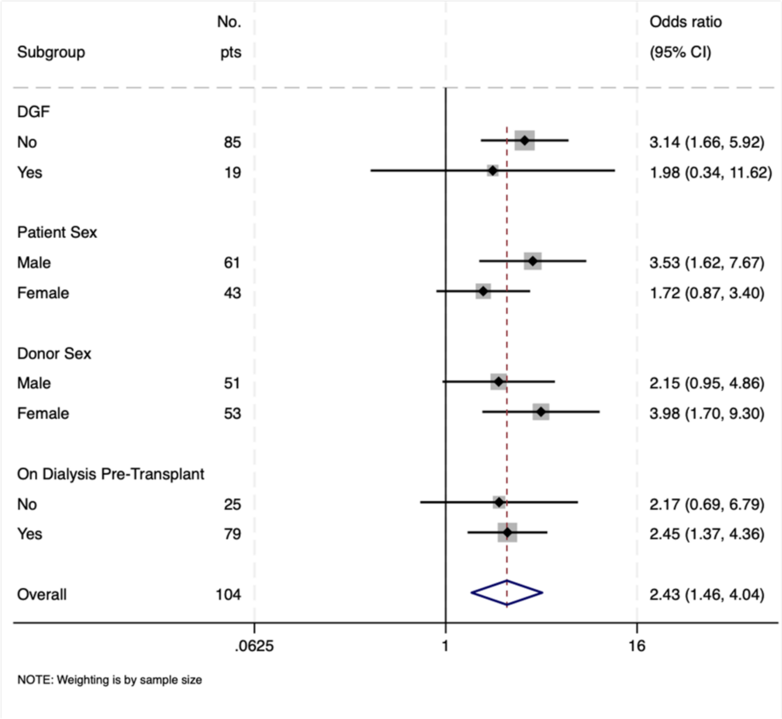

Figure S5: Unadjusted subgroup analyses of A) the association between day 3 API and mGFR and B) the odds for mGFR > 60 at 12 months post-transplant. API: urine ammonium-pH index. mGFR: measured glomerular filtration rate.
